# TACE versus TARE for patients with hepatocellular carcinoma: Overall and individual patient level meta analysis

**DOI:** 10.1002/cam4.5125

**Published:** 2022-08-09

**Authors:** Andrew M. Brown, Ihab Kassab, Marco Massani, Whitney Townsend, Amit G. Singal, Cigdem Soydal, Laura Moreno‐Luna, Lewis R. Roberts, Vincent L. Chen, Neehar D. Parikh

**Affiliations:** ^1^ Division of Gastroenterology and University of Michigan Ann Arbor Michigan USA; ^2^ Ospedale Regionale Treviso Treviso Italy; ^3^ Division of Digestive and Liver Diseases University of Texas Southwestern Dallas Texas USA; ^4^ Department of Nuclear Medicine Ankara University Medical School Ankara Turkey; ^5^ Division of Gastroenterology and Hepatology Mayo Clinic Rochester Minnesota USA

**Keywords:** HCC, locoregional therapy, TACE, Y‐90

## Abstract

**Background:**

Transarterial radioembolization (TARE) is increasingly used as an alternative to transarterial chemoembolization (TACE) for the treatment of hepatocellular carcinoma (HCC). We aimed to perform an overall and individual patient data (IPD) meta‐analysis of studies comparing TACE and TARE.

**Methods:**

We performed a systematic literature search using pre‐specified keywords with the aid of an informationist for articles from inception to 3/2020. The primary endpoint was overall survival (OS), and the secondary endpoint was time to progression (TTP).

**Results:**

Seventeen studies met inclusion criteria with 2465 unique patients, with one randomized trial, 4 prospective studies and 12 retrospective studies. Barcelona Clinic Liver Cancer (BCLC) stage B (42.8%) was the most common stage followed by BCLC A (30.3%) and BCLC C (29.0%). There was no difference in OS between the two modalities (−0.55 months, 95% CI −1.95 to 3.05). In three studies with available TTP data, TARE resulted in a longer TTP than TACE (mean TTP 17.5 vs. 9.8 months; mean TTP difference 4.8 months, 95% CI 1.3–8.3 months). IPD‐level meta‐analysis of 311 patients from three studies showed no difference in overall OS between the two modalities including among subgroups stratified by tumor stage and liver function. Limitations of the current literature include inconsistent length of follow‐up, inconsistency in response criteria, and safety reporting.

**Conclusions:**

Current data suggest TARE provides significantly longer TTP than TACE, although the two treatments do not significantly differ in terms of OS. Given limitations of the current data, there is rationale for prospective studies comparing these modalities.

## Graphical abstract

In this overall and individual level meta‐analysis, patients with hepatocellular carcinoma treated with transarterial chemoembolization and transarterial radioembolization (TARE) showed similar survival. Patients treated with TARE however, had a longer time to tumor progression.

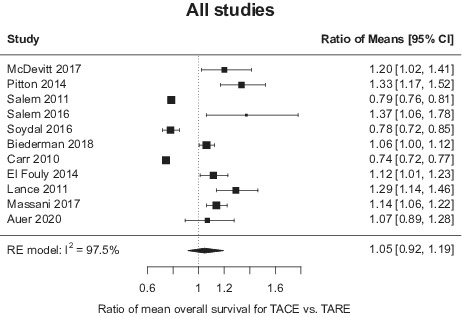

## 1 INTRODUCTION

Hepatocellular carcinoma (HCC) is the fourth leading cause of cancer related to mortality worldwide, largely owing to ineffective early detection strategies, competing risk from comorbid cirrhosis, and historical lack of effective therapies for intermediate and advanced stage disease.^1^ Curative therapies are typically reserved for patients with early‐stage HCC, resulting in >70% 5‐year survival, whereas intermediate and advanced disease have marked decrements in survival.^2^

Furthermore, there are no well‐established treatment algorithms, with significant variation in treatment application.^3^ This is due in part to the lack of head‐to‐head data comparing many treatment modalities leading to variance in treatment allocation. For example, in patients with early intermediate‐stage disease, transarterial chemoembolization (TACE) and transarterial radioembolization (TARE) are two commonly used loco‐regional therapies (LRT) which aim to prolong survival by slowing tumor progression, or to bridge to more definitive therapies. However, there is a lack of robust comparative effectiveness data comparing these treatments.

TACE has the highest quality of evidence among LRTs, supported by several randomized controlled trials.^4^, ^5^, ^6^ TARE recently attained Food and Drug Administration premarket approval with publication of recent large observational study showing excellent efficacy in the treatment of unifocal HCC in a multicenter cohort^7^; however, due to lack of robust controlled data, TARE has not been widely adopted as a frontline treatment in guidelines.^8^, ^9^ A single center randomized controlled trial with 45 patients comparing these modalities showed no difference in overall survival (OS), but showed longer time to progression with TARE, although generalizability remains limited due to the overall design and sample size.^10^ Existing observational data have been similarly limited by sample size, patient selection, and lack of correlates for treatment efficacy. While other meta‐analyses comparing TACE and TARE have been completed, we lack multicenter individual level data comparing TARE and TACE and correlates of survival. Our aim was to perform a meta‐analysis, including individual patient data, comparing TACE and TARE to determine the comparative effectiveness of these treatments.

## 2 METHODS

### 2.1 Search strategy

The Preferred Reporting Items for Systematic Reviews and Meta‐Analyses (PRIMSA) guidelines were used to guide study selection and data collection.^11^ Additionally, the study was registered in PROSPERO (CRD42019129117) prior to its initiation. A systemic literature search using PubMed, Medline, EMBASE, Scopus, Web of Science, and ClinicalTrials.gov was performed using pre‐specified keywords with the aid of an informationist for articles from inception to March 2020. The search strategy was included in Appendix S1. Inclusion criteria were studies that directly compared TACE and TARE for the treatment of HCC. Exclusion criteria included study of the use of additional treatment modalities (e.g. other locoregional or systemic therapies), liver tumors besides primary HCC, and studies published only in abstract form. Only full text English language studies were included. W combined modalities of drug eluting bead (DEB) TACE and conventional TACE (cTACE) and the modalities of therasphere TARE and SIRS‐Sphere TARE due to the lack of data showing significant differences in outcomes between modalities.^12^, ^13^

### 2.2 Data extraction

The search yielded 1784 unique articles that were screened for inclusion by two independent reviewers (AB and IK). Conflicts were resolved with the assistance of a third reviewer (NP). Studies to be included were more closely analyzed by the authors and selected for appropriateness for data extraction. Data were extracted by each reviewer using standardized forms. Forms collected demographic, liver function, cancer staging, unadjusted survival, time to progression data, and adverse events when available. Study quality assessment was performed with Newcastle‐Ottawa Scale (NOS).

### 2.3 Outcomes

The primary endpoint was overall survival (OS), and the secondary endpoint was time to progression (TTP).

### 2.4 Individual patient data (IPD) meta‐analysis

Authors of all included studies were contacted for data sharing to include patient‐level data to allow IPD meta‐analysis, with the authors of three studies responding. These studies included papers by Moreno‐Luna et al.,^14^ Soydal et al.,^15^ and Massani et al.^16^ Prior to data transfer, we obtained University of Michigan and local site institutional review board approval, and deidentified data were transferred using data use agreements.

### 2.5 Statistical analysis

OS and TTP were reported in four different ways: (1) mean and standard deviation, (2) mean and standard error, (3) median and interquartile range, or (4) median and 95% confidence interval. To facilitate meta‐analysis, we harmonized all outcome reporting into mean and standard deviation per (1). For (2), we used reported mean and calculated $\mathrm{SD}=\mathrm{SE}*\sqrt{n}$. For (3), we estimated mean as $\frac{T_{Q1}+T_{\mathrm{med}}+T_{Q3}}{3}$ and standard deviation as $\frac{T_{Q3}-T_{Q1}}{2\Phi^{-1}\left(\frac{0.75n-0.125}{n+0.25}\right)}$, where T represents time (OS or TTP), Q1 represents the 25th percentile time, med the median time, Q3 the 75th percentile time, $\Phi^{-1}\left(x\right)$ the upper x‐th percentile, and n the number of patients receiving that specific treatment. For (4), we estimated mean as $\frac{T_{L}+2*T_{\mathrm{med}}+T_{H}}{4}$ and standard deviation as $\frac{T_{H}-T_{L}}{3.92}$, where *T* represents time, either OS or TTP, L represents the lower bound of the confidence interval, med the median time, and H the upper bound of confidence interval.^17^

Overall meta‐analysis was performed comparing TACE and TARE using a random effects model incorporating mean OS/TTP, standard deviation, and n for TACE and TARE recipients. We evaluated mean difference of both OS and TTP. Preplanned subgroup analyses stratifying studies by study quality. We assessed publication bias visually using a funnel plot and numerically with an Egger test.

IPD meta‐analysis was performed using data from three studies. The primary outcome was OS, as data on TTP were not available in the studies in which IPD were available. We generated Cox proportional hazard survival models rather than comparing difference in mean survival as these models are more informative. We conducted pre‐specified subgroup analyses based on BCLC stage and CP class, in which we separately evaluated hazard ratio for OS in each study and subgroup, and then meta‐analyzed across studies and within each subgroup (cluster random effects meta‐analysis) using a random effects meta‐analysis similar to as described above. Finally, we generated in each cohort a multivariable Cox proportional hazard survival model with outcome of OS and predictor of treatment type (TARE vs. TACE), adjusting for age, sex, CP class, and BCLC stage, and meta‐analyzed across the three cohorts as above.

*p* < 0.05 was used for significance in all analyses. All analyses were performed in R version 3.5.1.

## 3 RESULTS

### 3.1 Overall cohort characteristics

In total, our initial search revealed 1784 studies, 17 of which met inclusion criteria including 2465 unique patients (Figure S1). Twelve of the included studies were retrospective cohort studies and the remainder consisted of one randomized trial and four prospective cohort studies.^10^, ^14^, ^15^, ^16^, ^18^, ^19^, ^20^, ^21^, ^22^, ^23^, ^24^, ^25^, ^26^, ^27^, ^28^, ^29^, ^30^ The mean patient age was 62.1 years, and the majority were male (77.0%) and white (74.3%). Approximately two‐thirds of patients had compensated cirrhosis (Child Pugh [CP] A 65.4%) and the remainder were decompensated (CP B: 30.9%; CP C: 2.1%). Barcelona Clinic Liver Cancer (BCLC) stage B (42.1%) was the most common stage followed by BCLC C (29.0%) and BCLC A (30.3%) (Table 1).

**TABLE 1:** Descriptive statistics of overall meta‐analysis stratified by treatment type

Parameter	Overall (*n* = 2465)	TACE (*n* = 1657)	TARE (*n* = 808)
Mean age (years)	62.1	60.8	66.5
Male (%)	77.0	77.0	76.9
Race/Ethnicity			
White (%)	74.3	71.8	76.9
Black (%)	12.0	11.7	12.3
Hispanic (%)	6.8	8.0	5.6
Asian (%)	8.7	9.2	8.3
Other race (%)	6.9	10.0	3.6
Etiology of cirrhosis			
Alcohol (%)	27.2	26.7	28.2
Hepatitis C (%)	31.2	29.4	35.2
Hepatitis B (%)	10.7	11.5	8.7
Non‐alcoholic steatohepatitis (%)	6.0	5.7	6.2
Other etiology (%)	22.8	23.0	22.6
Child‐Pugh class			
A (%)	65.4	66.0	64.6
B (%)	30.9	29.2	33.3
C (%)	2.1	2.0	2.3
Barcelona Clinic Liver Cancer Stage			
A (%)	30.3	33.1	27.4
B (%)	42.8	41.8	43.8
C (%)	29.0	27.3	30.7
D (%)	3.1	3.7	2.5

Abbreviation: BCLC, Barcelona Clinic Liver Cancer.

Patients receiving TARE were significantly more likely to have chronic hepatitis C as the etiology of their liver disease (35.2% vs. 29.4%; *p* = 0.008), but otherwise there were no differences between the groups.

### 3.2 TACE and TARE modalities

Conventional TACE (cTACE) was the most commonly used modality in the included studies (8/17). Five of the studies used drug‐eluting bead transarterial chemoembolization (DEB‐TACE), while three studies used both cTACE and DEB‐TACE. One study used TACE with degradable starch microspheres (DSM). Each study had a mix of lobar treatment and segmental treatments.

With regard to TARE, the majority of the studies (10/17) used TheraSphere while six studies used SIR‐sphere, and one study used both (Table 2). Notably, no study specified the use of individual dosimetry for TARE delivery, although four used selective catheterization of the tumor vessels for treatment delivery.

**TABLE 2:** Hepatocellular carcinoma treatment modalities used by the included studies

Study	Year	TACE *n*	TACE modality	TACE treatment	TARE *n*	TARE modality	TARE treatment
Akinwande^24^	2016	28	DEB‐TACE	Lobar and selective	20	TheraSphere	Lobar
Akinwande^21^	2015	291	DEB‐TACE	Lobar and selective	67	TheraSphere	Lobar
Auer^30^	2021	18	DSM‐TACE	Lobar	18	SIR‐Sphere	Lobar
Biederman^22^	2018	877	DEB‐TACE	Selective	534	TheraSphere	Lobar
Carr^29^	2010	691	cTACE	Lobar and selective	99	TheraSphere	Lobar
El Fouly^20^	2014	42	cTACE	Selective	44	TheraSphere	Lobar
Kooby^19^	2010	44	cTACE	Selective	27	SIR‐Sphere	Lobar
Lance^18^	2011	35	cTACE and DEB‐TACE	Selective	38	TheraSphere and SIR‐Sphere	Lobar and Selective
Massani^16^	2017	82	cTACE and DEB‐TACE	Lobar and Selective	39	SIR‐Sphere	Lobar
McDevitt^27^	2017	24	DEB‐TACE	NS	26	TheraSphere	NS
Moreno‐Luna^14^	2012	55	cTACE	Lobar and Selective	61	TheraSphere	Lobar
Padia^26^	2017	77	cTACE and DEB‐TACE	Selective	101	TheraSphere	Selective
Pitton^25^	2014	12	DEB‐TACE	Selective	12	SIR‐Sphere	Lobar
Salem^23^	2011	122	cTACE	NS	123	TheraSphere	NS
Salem^10^	2016	21	cTACE	NS	24	TheraSphere	NS
She^28^	2014	16	cTACE	Lobar and Selective	16	SIR‐Sphere	Selective
Soydal^15^	2016	40	cTACE	Selective	40	SIR‐Sphere	Selective

Abbreviations: cTACE, conventional transarterial chemoembolization; DEB, Drug eluting bead; DSM, degradable starch microsphere; NS, not specified; TACE, Transarterial chemoembolization; TARE, transarterial radioembolization.

### 3.3 Outcomes

**FIGURE 1:**
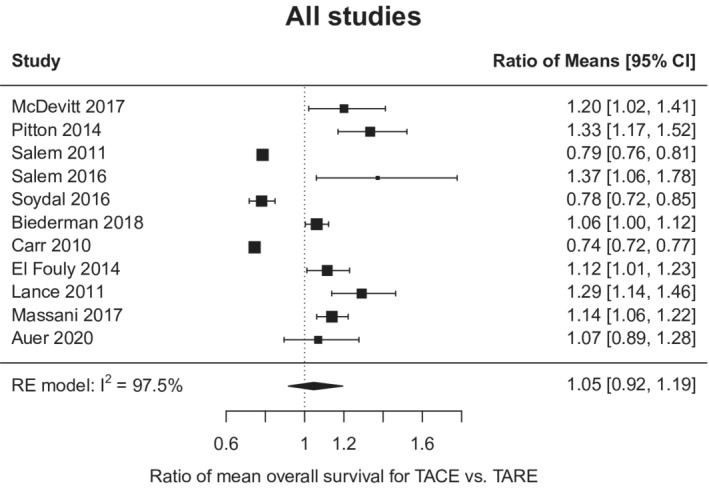
Forest plot of mean overall survival for transarterial chemoembolization (TACE) vs transarterial radioembolization (TARE). RE, random effects

**FIGURE 2:**
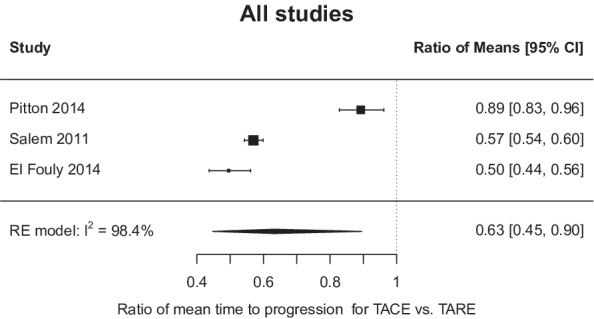
Forest plot of mean time to progression for transarterial chemoembolization (TACE) vs transarterial radioembolization (TARE). RE, random effects

There was no difference in OS between the two modalities with absolute difference −0.55 months, 95% CI −1.95 to 3.05 (for TARE relative to TACE) (Figure 1); however, there was notable heterogeneity among the studies (*I* ^2^ 97.5%; *p* < 0.001). In the three studies with available TTP data, TARE resulted in a significantly longer TTP than TACE (mean TTP 17.5 vs. 9.8 months; mean TTP difference 4.8 months, 95% CI 1.3–8.3 months) (Figure 2). Similarly, heterogeneity was again seen among the studies reporting the TTP outcome (*I* ^2^ > 97%; *p* < 0.001).

### 3.4 Individual level meta‐analysis

**FIGURE 3:**
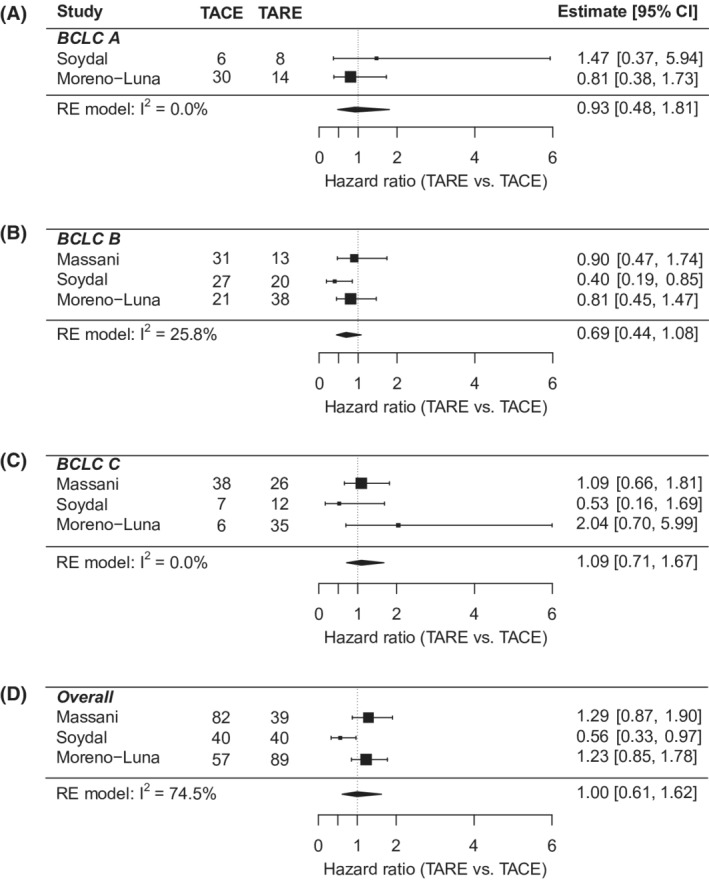
Individual‐level meta‐analysis forest plots for (A) Barcelona Clinic Liver Cancer stage (BCLC) A, (B) B, (C) C; (D) overall. Hazard ratio <1 implies TARE is favored and >1 implies TACE is favored. RE, random effects; TACE, transarterial chemoembolization; TARE, transarterial radioembolization

**FIGURE 4:**
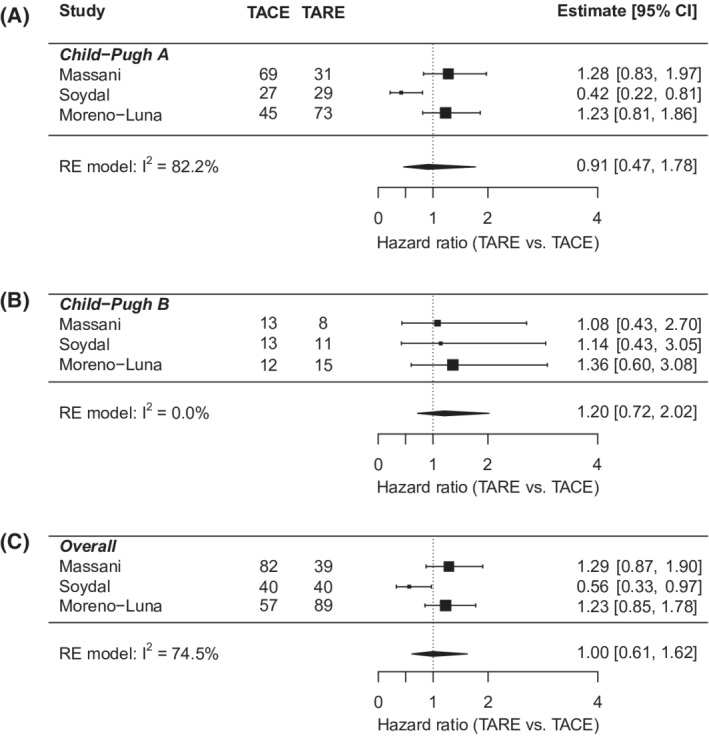
Individual‐level meta‐analysis forest plots for (A) Child Pugh A, (B) Child Pugh B, (C) overall. Hazard ratio <1 implies TARE is favored and >1 implies TACE is favored. RE, random effects. TACE, transarterial chemoembolization. TARE, transarterial radioembolization

Individual‐level meta‐analysis included 311 patients, 143 in the TACE group and 168 in the TARE group (Table 3). Mean age was 67.8 years, 81% were male, and 88.5% were white. 61% of patients were BCLC stage A. And 83.2% had CP A liver function, while 16.8% had CP B. The cohort characteristics were similar between the TACE and TARE groups, except for a higher proportion of BCLC A patients in the TACE group (26.4% vs 12.2%; *p* = 0.012). Overall hazard ratio showed no difference in OS between TARE and TACE (HR: 0.90; 95% CI: 0.70–1.16). The results were consistent in key subgroups stratified by BCLC and CP class (Figures 3 and 4). In multivariate analysis (Table S1), worse OS was associated with male sex (vs. females) (HR: 1.43; 95% CI: 1.07–1.92); Child Pugh B/C cirrhosis (vs. CP A) (HR: 1.36; 95% CI: 1.02–1.82); and more advanced stage HCC (BCLC B vs. A; HR: 1.58; 95% CI: 1.13–2.21; BCLC C/D vs. A; HR: 2.08; 95% CI: 1.46–2.95).

**TABLE 3:** Descriptive statistics of individual‐level meta‐analysis stratified by treatment type

Parameter	Overall	TACE (*n* = 179)	TARE (*n* = 168)	*p*‐value
Mean age (years)	67.8	68.6	66.6	0.86
Male (%)	81.3	81.3	81.3	1.00
Race/Ethnicity				
White (%)	88.5	88.0	89.0	1.00
Black (%)	2.1	0.0	4.0	0.40
Hispanic (%)	3.1	2.0	4.0	0.93
Other race (%)	6.4	9.0	4.0	0.47
Etiology of cirrhosis				
Alcohol (%)	36.0	36.6	35.2	0.93
Hepatitis C (%)	21.6	23.5	18.9	0.50
Hepatitis B (%)	13.2	13.4	12.8	1.00
Non‐alcoholic steatohepatitis (%)	12.7	18.0	8.0	0.18
Other etiology (%)	29.2	24.2	36.0	0.069
Child‐Pugh class				
A (%)	83.2	82.5	84.1	0.88
B (%)	16.8	17.5	15.9	
Barcelona Clinic Liver Cancer Stage				
A (%)	20.4	26.4	12.2	0.012
B (%)	46.6	44.2	49.8	0.38
C (%)	31.9	30.8	33.2	0.73

### 3.5 Publication bias and study quality

There was no evidence of publication bias for mean differences for either OS or TTP based on Egger's regression test and rank correlation test (Figure S2).

The mean total quality score for the included studies was 7.2 on NOS. Sixteen studies (84%) were high quality (NOS ≥ 7) while one study was medium quality (NOS <7) (Table S2). There were only two studies classified as medium‐quality. When only high‐quality studies were included, OS with TARE was not significantly higher than with TACE (mean difference 0.27 months; 95% CI: −2.43 to 2.97; *I* ^2^: 98.1%.) (Figure S3).

### 3.6 Adverse events

The included studies featured a wide range of adverse events (AE) data. Only four studies reported data on AEs; however, there was significant heterogeneity in the reporting. Any AE associated with TACE patients was 10.8–73%, while any AE for TARE patients was 10%–44%.^18^, ^19^, ^21^, ^24^ In those same studies, grade 3–4 AEs were 4.5%–36% and 4%–30% for patients receiving TACE and TARE, respectively.^18^, ^19^, ^21^, ^24^ One study reported nausea and vomiting rates to be 16.5% versus 55%^30^ for TACE versus TARE; while another reported 38% versus 0% for TACE versus TARE.^20^ Two studies reported higher rates of abdominal pain in TACE (73%–83%) vs TARE (5%–33%).^20^, ^27^ Rates of diarrhea were 21% versus 0% in TACE versus TARE in one study.^10^ One study described a higher rate of post‐embolization syndromes in TACE (20%) compared to TARE (2.6%).^18^

## 4 DISCUSSION

In our updated meta‐analysis, we have demonstrated lack of a difference in overall survival in patients receiving TACE versus TARE; however, time to HCC progression was significantly longer in patients receiving TARE therapy. These findings are concordant with the only randomized data comparing TACE and TARE.^10^ In an individual meta‐analysis, we were able to confirm the lack of superiority of either modality for overall survival in subgroups of patients stratified by tumor stage and liver function. Notably, TARE had similar associated survival outcomes despite having a higher proportion of patients with more advanced stage liver disease (CP B) and advanced stage HCC (BCLC C). While there have been previous published meta‐analyses comparing these modalities,^31^, ^32^, ^33^ our study includes several additional contemporaneous studies, in addition to individual level meta‐analysis including data including 311 patients.

In the subgroup analyses, there was no difference in overall survival in patients regardless of the modality received. In studies stratifying TACE or TARE by BCLC class, there is consistent decrements in survival with advancing stages. Similarly, several studies have shown the significant decrements in TACE and TARE effectiveness and safety in CP B and C disease. TARE has also been compared to sorafenib in BCLB B and C disease in two randomized trials and was not associated with a survival benefit,^34^, ^35^ but was associated with superior quality of life and appears to be cost‐effective.^36^ In the multivariate analysis of TACE versus TARE from the individual level meta‐analysis, several known correlates of worse survival were significant, including liver function and tumor burden. When controlling for these, TACE and TARE still had similar survival. With the advent of more efficacious therapies for unresectable HCC, the utility of locoregional therapies in more advanced stage HCC deserves further study. While previous adjuvant therapy trials involving TACE and sorafenib in unresectable disease have not shown a survival benefit, there are several ongoing trials pairing inter‐arterial therapies with immunotherapy‐based systemic regimens. However, the majority of these trials are in combination with TACE due to the limited data behind TARE.^37^

Nevertheless, TACE and TARE remain primary treatment options for a significant proportion of patients with HCC and our analysis supports the efficacy of both. TARE was associated with an increased TTP compared to TARE in a subset of the included studies; however, there are notable deficiencies in radiographic interpretation of tumor progression after radiation therapy that may have contributed to this finding.^38^ While this deserves further scrutiny due to the observed heterogeneity in the data and nonstandardized radiographic interpretation across studies, the increased TTP is a meaningful outcome for a bridging population while awaiting more definitive therapies, such as liver transplantation.

Our study had several strengths and limitations. First, TACE and TARE technique for administration and patient selection were not standardized across the studies. Most of the studies included were retrospective in design, thus bias such as confounding by indication, differences in imaging interpretation, or unmeasured confounders may have contributed to the results of the analysis. Furthermore, treatment with TARE has evolved with several recent studies including personalized dosimetry as the most effective method for TARE delivery; however, most of the included studies utilized lobar treatment or standardized dosimetry.^37^, ^39^ Similarly, TACE is increasingly delivered in a selective fashion, whereas several of the studies included lobar delivery of TACE. There was also significant heterogeneity in the meta‐analysis results, likely reflecting the differences in study design, patient selection, and treatment administration, however we did confirm the primary findings of the study in the individual level meta‐analysis. While our study focused on efficacy, we were limited in our ability to adequately compare the safety profiles of the treatments due to lack of consistent inclusion of safety data in the included studies. The safety data we were able to derive from the studies indicated a trend toward favorable adverse event profile for TARE compared to TACE. This is consistent with the limited data available on quality of life after TACE and TARE treatment.^40^, ^41^ Finally, we were unable to control for therapies received prior to and after the TACE or TARE in the included studies and differences in the follow‐up care of these patients may have been mediators of overall survival. The survival reporting was heterogeneous among studies as well, with inconsistent reporting of survival using adjusted HR or censored Kaplan Meier survival. In a subgroup analysis of studies reporting HR only, the results were similar. These weaknesses are balanced by the strengths of an updated comprehensive meta‐analysis and systematic review including thousands of patients showing consistent effects across several subgroup analyses and individual patient level meta‐analysis.

## 5 CONCLUSIONS

Current data suggest TARE can provide significantly longer TTP than TACE, although the two treatments do not significantly differ in terms of overall survival in both our overall and individual patient level meta‐analysis. Safety profiles appeared to favor TARE; however, these data deserve further prospective confirmation. Given the limitations of the current data, there is rationale for comparing these modalities in larger prospective analyses to allow granular comparison of survival, progression, and safety data.

## AUTHOR CONTRIBUTIONS

Parikh is the guarantor of this article. Roles: (a) Concept: Parikh; (b) Analysis: Parikh, Chen; (c) Data acquisition: All authors; (d) Writing: Parikh, Brown, Kassab, Singal; (e) Critical revision: All authors.

## FUNDING INFORMATION

Dr. Singal's research is conducted with support from National Institutes of Health U01 CA230694, R01 MD12565. Dr. Parikh's research is conducted with support from National Institutes of Health U01 CA230669. The content is solely the responsibility of the authors and does not necessarily represent the official views of the National Institutes of Health. The funding agencies had no role in design and conduct of the study; collection, management, analysis, and interpretation of the data; or preparation of the manuscript.

## CONFLICT OF INTEREST

Brown: None. Kassab: None. Massani: None. Townsend: None. Singal: Served as a consultant or on advisory boards for Bayer, FujiFilm Wako Diagnostics, Exact Sciences, Roche, Glycotest, and GRAIL. Soydal: None. Moreno‐Luna: None. Roberts: Consults for AstraZeneca, MJH Life Sciences, and Clinical care options; he advises and received grants from Bayer, Exact Sciences, and Gilead; he advises GRAIL, Tavec, QED Therapeutics, Genentech, Envision, and Eisai and received grants from Ariad, BTG International, GylcoTest, RedHill, Ltd Pharma, and Wako Diagnostics. Chen: None. Parikh: Served as a consultant for Bristol Myers‐Squibb, Exact Sciences, Eli Lilly, and Freenome; has served on advisory boards of Genentech, Eisai, Bayer, Exelixis, Wako/Fujifilm; and has received research funding from Bayer, Target RWE, Exact Sciences, Genentech and Glycotest.

## ETHICS STATEMENT

The data included in this study were either publicly available or completely de‐identified and were exempt from Institutional Review Board approval.

## Supporting information


Appendix S1

## Data Availability

Data from this study were taken from the published literature which is publicly available. The individual meta‐analysis data can be attained by contacting the authors of the studies used in this analysis.
