# Supplementary material for: TACE versus TARE for patients with hepatocellular carcinoma: Overall and individual patient level meta analysis
Source: Cancer Med. 2022 Aug 9;12(3):2590–9. doi: 10.1002/cam4.5125 (PMC9939158; doi:10.1002/cam4.5125)

**Supplement for manuscript:** Systematic Review and Meta-Analysis: Transarterial Chemoembolization (TACE) versus Transarterial Radioembolization (TARE) for Patients with Hepatocellular Carcinoma

Search Strategy:

**Search strategy per database through March 2020.**

# PubMed

# ("Hepatocellular carcinoma"[tw] OR "Hepatocellular carcinomas"[tw] OR "Liver carcinoma"[tw] OR "Liver carcinomas"[tw] OR "Liver cell carcinoma"[tw] OR "Liver cell carcinomas"[tw] OR Hepatoma[tw] OR Hepatomas[tw] OR "Hepatocellular Cancer"[tw] OR "Hepatocellular cancers"[tw] OR HCC[tiab] OR "Carcinoma, Hepatocellular"[Mesh]) AND ("Transarterial Radioembolization"[tw] OR Radioemboliz*[tw] OR radioembolis*[tw] OR TARE[tw] OR "yttrium-90"[tw] OR "Y90"[tw] OR Yttrium[tw] OR "selective internal radiation therapy"[tw] OR SIRT[tw]) AND ("transarterial chemoembolization"[tw] OR TACE[tiab] OR Chemoemboliz*[tw] OR chemoembolis*[tw] OR Mytomycin[tw] OR "Chemoembolization, Therapeutic"[Mesh])

**Embase**

('Hepatocellular carcinoma':ti,ab OR "Hepatocellular carcinomas":ti,ab OR "Liver carcinoma":ti,ab OR "Liver carcinomas":ti,ab OR "Liver cell carcinoma":ti,ab OR "Liver cell carcinomas":ti,ab OR Hepatoma:ti,ab OR Hepatomas:ti,ab OR "Hepatocellular Cancer":ti,ab OR "Hepatocellular cancers":ti,ab OR HCC:ti,ab OR 'liver cell carcinoma'/exp) AND ('transarterial radioembolization':ti,ab OR radioemboliz*:ti,ab OR radioembolis*:ti,ab OR tare:ti,ab OR 'yttrium-90':ti,ab OR 'y90':ti,ab OR yttrium*:ti,ab OR 'radioembolization'/exp OR 'selective internal radiation therapy':ti,ab OR SIRT:ti,ab) AND ('transarterial chemoembolization':ti,ab OR tace:ti,ab OR chemoemboliz*:ti,ab OR chemoembolis*:ti,ab OR mytomycin:ti,ab OR 'chemoembolization'/exp)

**Scopus**

(TITLE-ABS-KEY({Hepatocellular carcinoma} OR {Hepatocellular carcinomas} OR {Liver carcinoma} OR {Liver carcinomas} OR {Liver cell carcinoma} OR {Liver cell carcinomas} OR Hepatoma OR Hepatomas OR {Hepatocellular Cancer} OR {Hepatocellular cancers} OR HCC OR {Carcinoma, Hepatocellular})) AND (TITLE-ABS-KEY({Transarterial Radioembolization} OR Radioemboliz* OR radioembolis* OR TARE OR {yttrium-90} OR Y90 OR Yttrium* OR {selective internal radiation therapy} OR SIRT)) AND (TITLE-ABS-KEY({transarterial chemoembolization} OR TACE OR Chemoemboliz* OR chemoembolis* OR Mytomycin))

**Web of Science Core Collection**

TOPIC: (("Hepatocellular carcinoma" OR "Hepatocellular carcinomas" OR "Liver carcinoma" OR "Liver carcinomas" OR "Liver cell carcinoma" OR "Liver cell carcinomas" OR Hepatoma OR Hepatomas OR "Hepatocellular Cancer" OR "Hepatocellular cancers" OR HCC OR "Carcinoma, Hepatocellular")) AND TOPIC: (("Transarterial Radioembolization" OR Radioemboliz* OR radioembolis* OR TARE OR "yttrium-90" OR Y90 OR Yttrium* OR "selective internal radiation therapy" OR SIRT)) AND TOPIC: (("transarterial chemoembolization" OR TACE OR Chemoemboliz* OR chemoembolis* OR Mytomycin))

**ClinicalTrials.gov**

Condition or Disease: Hepatocellular Carcinoma

Other Terms: (TARE OR radioembolization OR yttrium OR "selective internal radiation therapy" OR SIRT) AND (TACE OR chemoembolization OR mytomycin

Supplementary Tables:

**Supplemental Table 1. Multivariate analysis of factors associated with overall survival in the individual level meta-analysis (n=311). TACE, transarterial chemoembolization. TARE, transarterial radioembolization. BCLC, Barcelona Clinic Liver Cancer.**

|  | | **Hazard ratio (95% CI)** | **p-value** |
| --- | --- | --- | --- |
| **TARE (vs. TACE)** | | 0.90 (0.70-1.16) | 0.43 |
| **Age (per year)** | | 1.00 (0.98-1.01) | 0.46 |
| **Male sex (vs. female)** | | 1.43 (1.07-1.92) | 0.017 |
| **Child-Pugh class B-C (vs. A)** | | 1.36 (1.02-1.82) | 0.037 |
| **BCLC stage** | |  |  |
|  | **A** | (Referent) |  |
|  | **B** | 1.58 (1.13-2.21) | 0.0072 |
|  | **C/D** | 2.08 (1.46-2.95) | <0.001 |
| **Study** | |  |  |
|  | **Massani** | (Referent) |  |
|  | **Moreno-Luna** | 1.54 (1.16-2.04) | 0.0029 |
|  | **Soydal** | 1.18 (0.84-1.67) | 0.34 |

**Supplemental Table 2. Study quality assessment using Newcastle-Ottawa Scale (NOS)**

| **Author(s)** | **Year** | **Selection** | **Comparability** | **Outcome** | **Total quality score (maximum score of 9)** |
| --- | --- | --- | --- | --- | --- |
| Akinwande ^21^ | 2016 | 3 | 1 | 3 | 7 |
| Akinwande ^18^ | 2015 | 2 | 1 | 3 | 6 |
| Auer ^27^ | 2021 | 3 | 2 | 3 | 8 |
| Biederman ^19^ | 2018 | 3 | 1 | 3 | 7 |
| Carr ^26^ | 2010 | 3 | 1 | 3 | 7 |
| El Fouly ^17^ | 2014 | 3 | 2 | 3 | 8 |
| Kooby ^16^ | 2010 | 3 | 1 | 3 | 7 |
| Lance ^15^ | 2011 | 3 | 2 | 3 | 8 |
| Massani ^13^ | 2017 | 2 | 1 | 3 | 6 |
| McDevitt ^24^ | 2017 | 3 | 2 | 3 | 8 |
| Moreno-Luna ^11^ | 2012 | 3 | 2 | 3 | 8 |
| Padia ^23^ | 2017 | 3 | 1 | 3 | 7 |
| Pitton ^22^ | 2014 | 3 | 2 | 3 | 8 |
| Salem ^20^ | 2011 | 3 | 1 | 3 | 7 |
| Salem ^9^ | 2016 | 2 | 2 | 3 | 7 |
| She ^25^ | 2014 | 2 | 2 | 3 | 7 |
| Soydal ^12^ | 2016 | 3 | 2 | 3 | 8 |
| **Average total score** |  |  |  |  | 7.29 |

**Note:** selection: representativeness of studies (maximum score of 4). Comparability: comparability of studies based on the design or analysis (maximum score of 2). Outcome: assessment of outcome and follow-up (maximum score of 3).

# Figures

Supplementary Figures:

Supplementary Figure Legend:

Supplementary Figure 1. PRISMA flow chart of the search strategy results for meta-analysis comparing TACE and TARE for the treatment of HCC

Supplementary Figure 2. Funnel plots for publication bias for overall survival and time to progression

Supplementary Figure 3. Subgroup analysis of high quality studies comparing TACE and TARE

**Supplementary Figure 1. PRISMA Flowchart**

## Screening

## Included

## Eligibility

## Identification

Additional records identified through other sources
(n = 0)

Records after duplicates removed
(n = 1784)

Records screened
(n = 1784)

Records excluded
(n = 1698)

Full-text articles excluded: Did not meet inclusion criteria (n=67) or overlapping cohorts (n=2)

Studies included in quantitative synthesis (meta-analysis)
(n = 17)

Records identified through database searching
(n = 3264)

Full-text articles assessed for eligibility
(n = 86)

Studies included in qualitative synthesis
(n = 17)

**Supplementary Figure 2: Overall survival stratified by high or low microvascular invasion (MVI). a) Mean Difference, b) Ratio of Means. MD, mean difference. RE, random effects.**

**
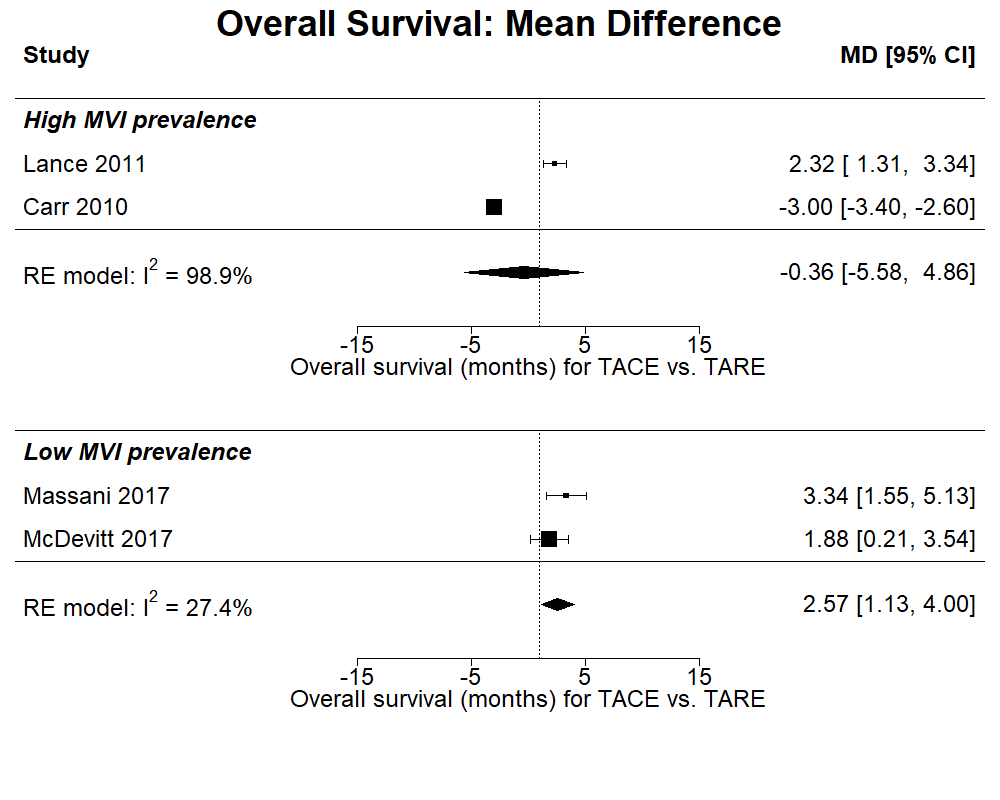
**

**
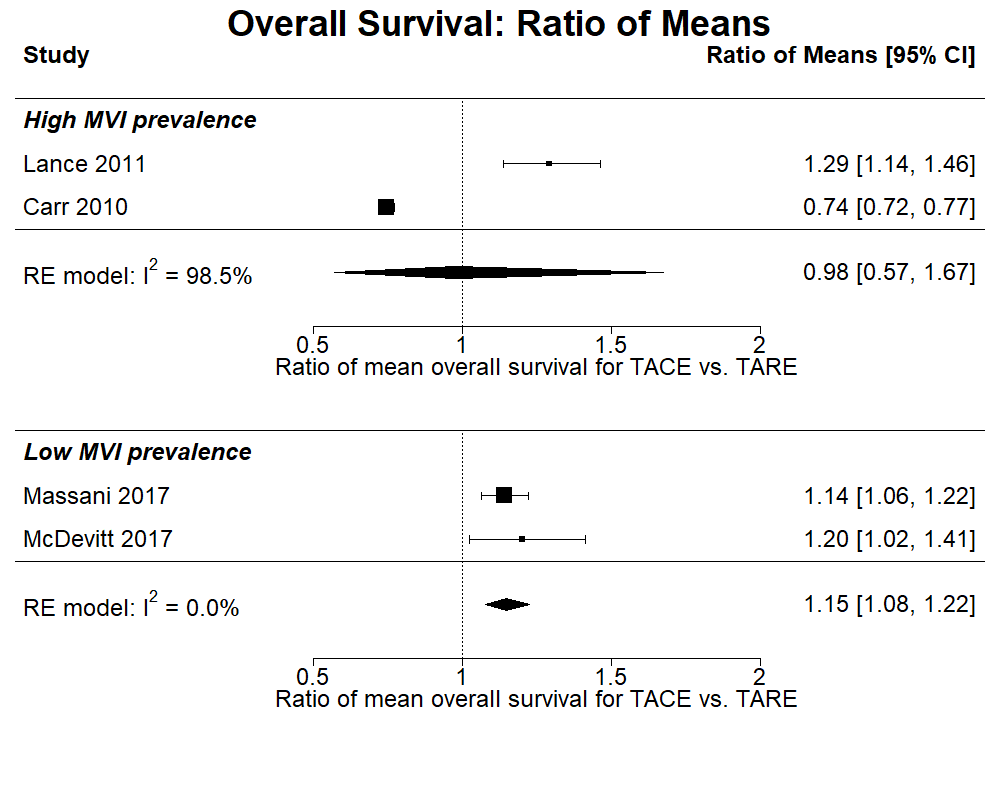
**

**Supplementary Figure 3. Funnel plots for publication bias for a) Overall survival, difference in means, b) overall survival, log ratio of means, c) time to progression, difference in means, and d) time to progression, log ratio of means. TACE, transarterial chemoembolization. TARE, transarterial radioembolizaion.**


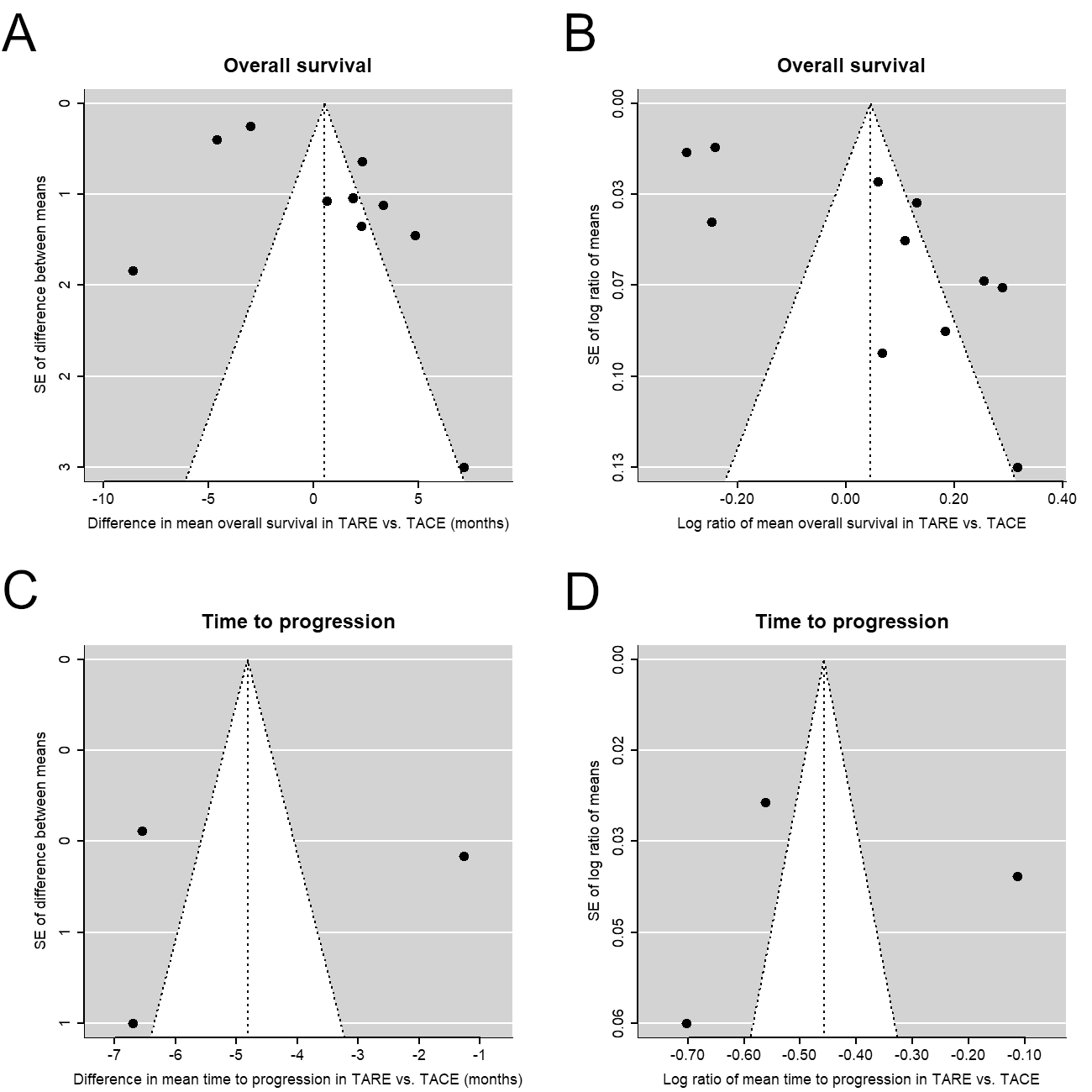


**Supplemental Figure 4a-b. Overall survival in high quality studies. a) Mean Difference, b) Ratio of Means. MD, mean difference. RE, random effects.**


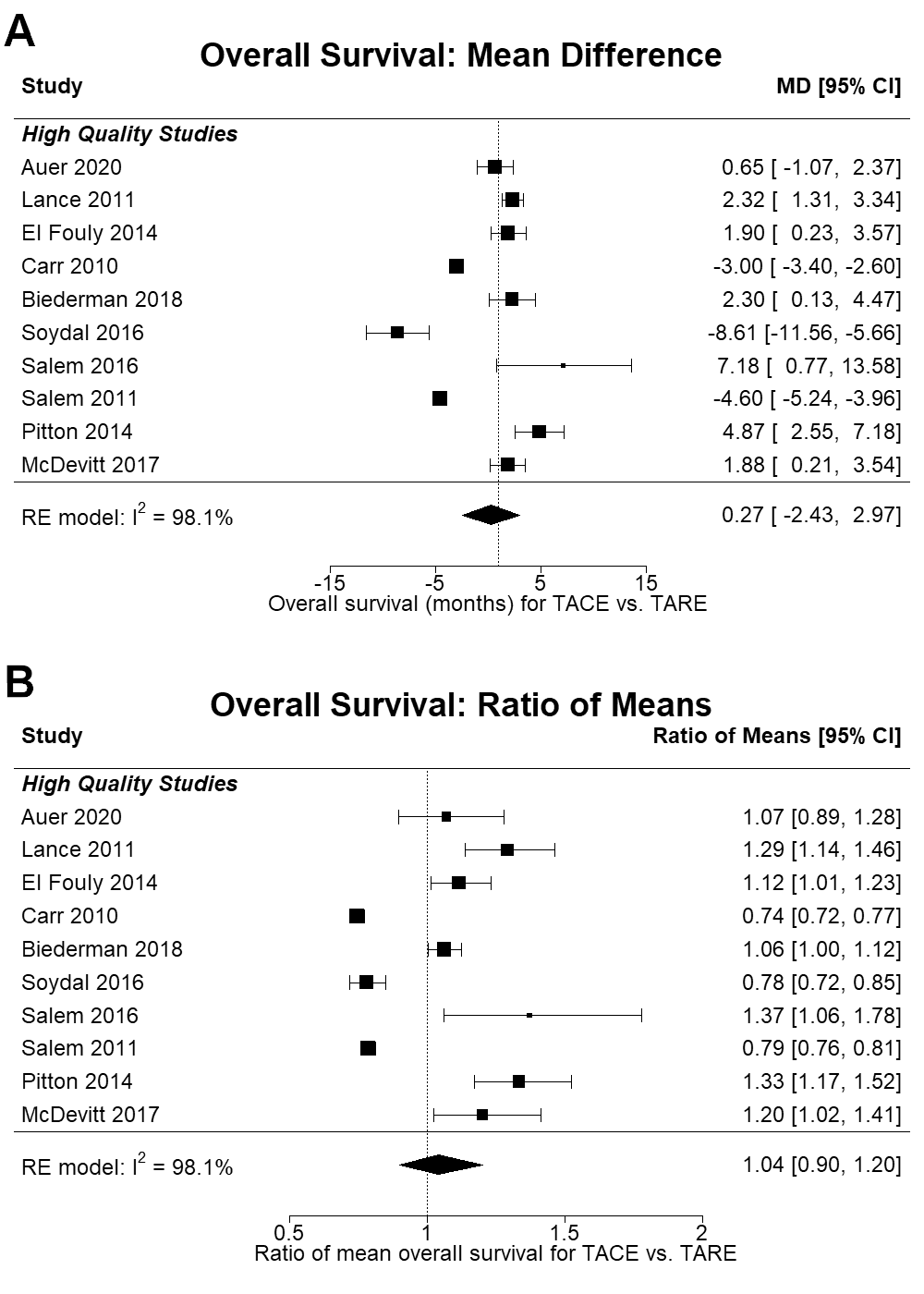

Supplement: Supplementary file 1 — Appendix S1 [file CAM4-12-2590-s001.docx]
